# Materials and Manufacturing Technologies Available for Production of a Pediatric Bioabsorbable Stent

**DOI:** 10.1155/2013/137985

**Published:** 2013-09-08

**Authors:** Ryan D. Alexy, Daniel S. Levi

**Affiliations:** Mattel Children's Hospital, University of California, Los Angeles, CA 90095, USA

## Abstract

Transcatheter treatment of children with congenital heart disease such as coarctation of the aorta and pulmonary artery stenosis currently involves the use of metal stents. While these provide good short term results, there are long term complications with their use. Children outgrow metal stents, obligating them to future transcatheter dilations and eventual surgical removal. A bioabsorbable stent, or a stent that goes away with time, would solve this problem. Bioabsorbable stents are being developed for use in coronary arteries, however these are too small for use in pediatric congenital heart disease. A bioabsorbable stent for use in pediatric congenital heart disease needs to be low profile, expandable to a diameter 8 mm, provide sufficient radial strength, and absorb quickly enough to allow vessel growth. Development of absorbable coronary stents has led to a great understanding of the available production techniques and materials such as bioabsorbable polymers and biocorrodable metals. Children with congenital heart disease will hopefully soon benefit from the current generation of bioabsorbable and biocorrodable materials and devices.

## 1. Introduction

Transcatheter treatment of children with congenital heart disease using angioplasty and stenting has evolved in recent years. While rare lesions are successfully treated with angioplasty, there is a significant incidence of acute vessel recoil and late aneurysm formation. The availability of metal stents can prevent recoil and lessen aneurysm formation. However, vessels containing a metal stent have a fixed vessel diameter, and this becomes a problem when placed in rapidly growing neonates or small children. The stented vessel is unable to grow with the child leading to a relative restenosis of the vessel. This obligates the child to require serial redilations, “unzipping procedures” or even surgical removal. A bioabsorbable stent (BAS) would allow for stent placement in small children without these issues, and it would revolutionize the treatment of many infants with common forms of congenital heart disease.

## 2. Need

Although bioresorbable materials have been used in medicine for several decades, these materials only recently have been utilized for pediatric transcatheter and surgical vascular and cardiac devices. Metallic stents are critical in the treatment of many types of congenital heart lesions such as coarctation of the aorta and pulmonary artery stenosis, though in most cases, these devices only need to serve as temporary scaffoldings. Their permanent presence can lead to complications such as thrombosis, late restenosis, and stent fracture. A biodegradable stent would provide the mechanical support required to prevent vessel recoil. The stent would then disappear as the vessel heals and no longer requires structural support. Once the stent absorbs, the vessel would be able to grow with child, eliminating the need for future surgery [1, 2]. Ideally the vessel would regain its vasoreactivity. The ABSORB trial of the Bioresorbable Vascular Scaffold (BVS) everolimus-eluting stent, a bioabsorbable stent developed for use in coronary arteries, showed restoration of a functionally normal endothelium at the stented site in some patients [3]. Even in the absence of growth in the target vessel a bioabsorbable stent would facilitate future intervention such as angioplasty and would allow for improved access to previously jailed side branches [4, 5]. Bioabsorbable stents are also compatible with noninvasive imaging such as MRI and CT angiography, and they would not hamper future treatment options [5–8]. The ideal bioabsorbable stent would provide enough radial force to resist recoil, would be acceptably flexible for deployment into the distal pulmonary arteries of a small child, and should absorb without creating a significant local inflammatory response or systemic toxicities [4, 5].

It has been hypothesized that structural vascular support from a pediatric BAS for three to six months will be sufficient for long term healing [1, 9]. The stent would then lose structural integrity in nine to 12 months, allowing for vessel growth. It should be low profile, requiring less than a 6F sheath for delivery [10]. The ideal pediatric stent length and diameter are 15 mm–20 mm and 6 mm–9 mm, respectively [10]. The diameter of stents being developed for coronary arteries is 3-4 mm (1), so they are too small for reliable treatment of most pulmonary artery stenosis and aortic coarctations.

Reduction or avoidance of stent restenosis is a huge priority in the treatment of coronary lesions with bioabsorbable stents, though this could be much less of a concern for treatment of congenital heart disease. Even in newborns, these vessels are much larger than coronary arteries and less prone to severe neointimal hyperplasia and restenosis. Thus, it is likely that bioabsorbable stents designed specifically for the aorta and pulmonary arteries in children with congenital heart disease will be more successful and will not require drug-eluting agents to be imbedded within the stent matrix. 

This paper will review the congenital heart lesions most commonly treated with stents, the materials and manufacturing techniques available for production of bioabsorbable stents, and the bioabsorbable stents currently under development and testing.

## 3. Coarctation of the Aorta

Coarctation is a narrowing of the aorta typically just after or close to the origin of the left subclavian artery. Many coarctations are associated with “ductal” tissue that constricts the lumen of the aorta as the ductus arteriosus closes. In some newborns, severe coarctation causes immediate congestive symptoms, left ventricular failure and can be life threatening. Mild coarctation can cause progressive hypertension, left ventricular hypertrophy, and claudication. With an incidence of 1.5 to 3 per 10,000 live births, coarctation is one of the most common forms of congenital heart disease [11].

**Figure 1:**
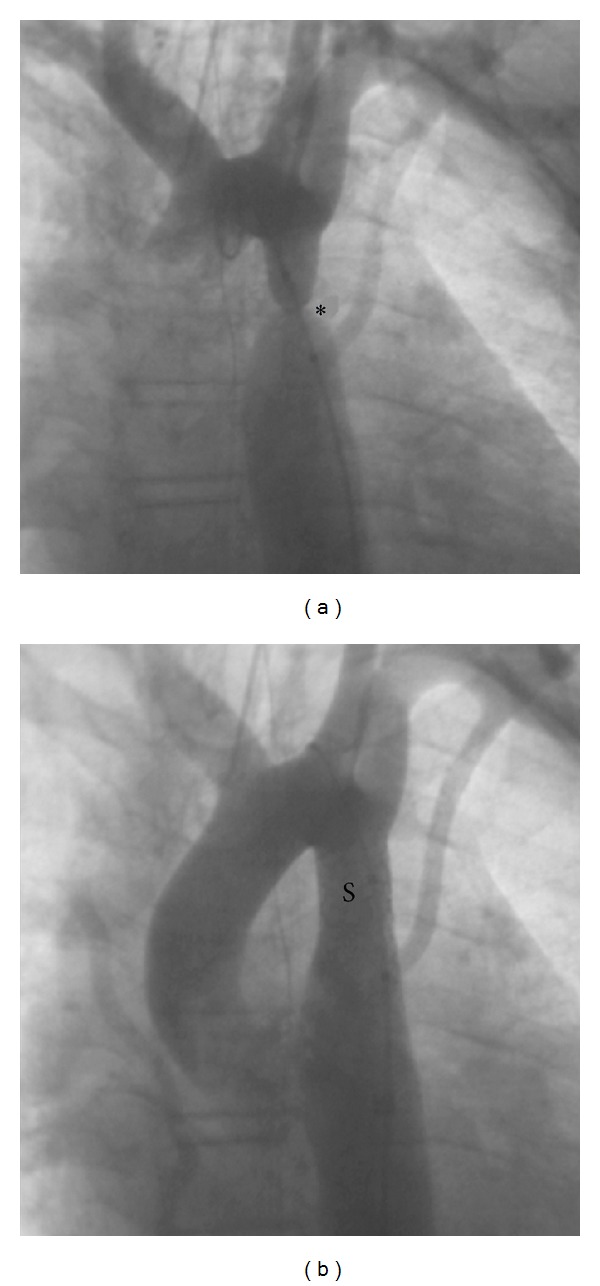
Coarctation (∗) before (a) and after (b) stent (S) placement. This procedure performed by our team provided complete resolution of this severe aortic stenosis.

**Figure 2:**
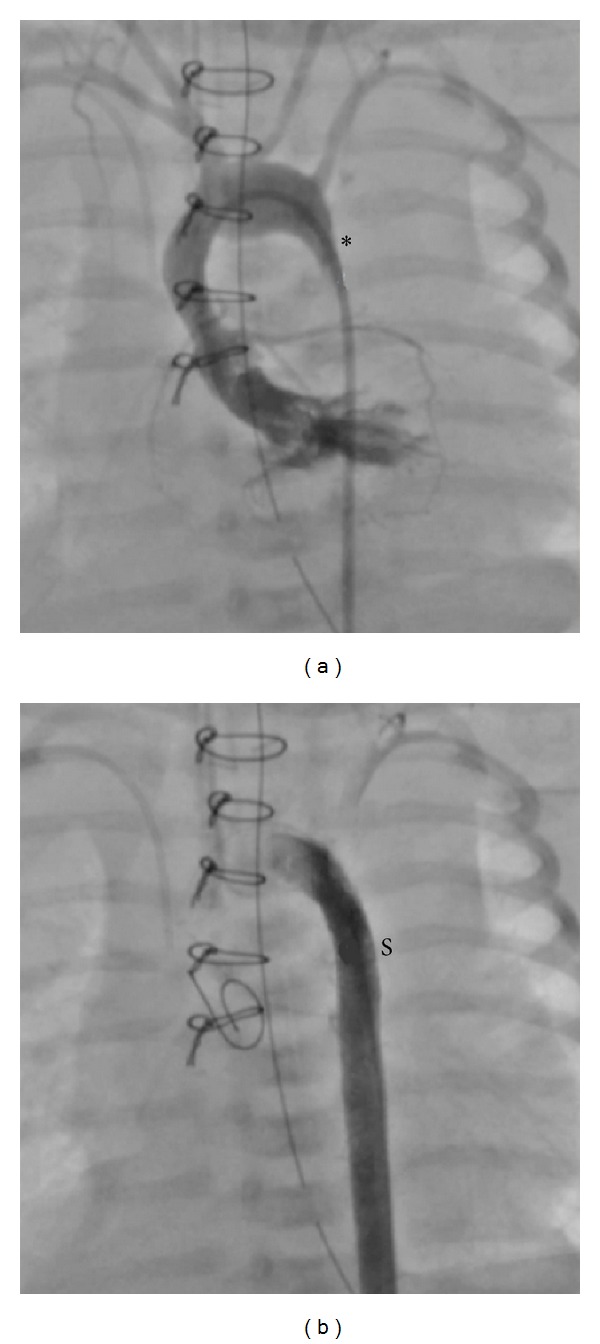
Newborn coarctation ((a), ∗) effectively treated by our team with a coronary stent ((b), S) in a very ill newborn. This demonstrates that coarctation in newborns can be effectively treated with a stent.

In older children, stenting of coarctation is very simple as the narrowing can be easily accessed and stented with bare metal stents from the femoral artery (Figure 1). Stenting in newborns and smaller children (<15 kg) can often be more technically challenging, but it is still able to be performed at relatively low risk to the patient, though surgery remains the gold standard due to the requirement that current BMS will need to be redilated at least 3x their initial implantation size to reach adult size as the child grows. Thus, stenting for coarctation in infants and smaller children is only performed as palliation in children deemed too sick for surgical intervention (Figure 2).

Unfortunately, surgical intervention for coarctation in newborns continues to have significant rates of mortality (2%), morbidity (up to 12–20%), and failure (recoarctation up to 18%) [12, 13]. A BRS would allow pediatric interventionalists to offer a very safe, effective, and minimally invasive approach to pediatric coarctation of the aorta in this high risk patient population.

In the optimal situation, a bioabsorbable coarctation stent would be implanted within the coarctation segment at the time of clinical presentation in the newborn or infant (as was the case for the BMS shown in Figure 2). The stent would provide complete relief of obstruction and would allow the aortic wall to remodel to an appropriate diameter (that of the stent) after which the stent would degrade. It is presumed that as the stent naturally degraded and absorbed, the vessel wall would continue to grow normally without the need for additional reintervention. An acceptable but less optimal alternative would be for stent implantation and degradation as above with a residual relative stenosis in the area of stenting. This stenosis could be then more easily and safely treated later with transcatheter methods.

## 4. Pulmonary Artery Stenosis

Most commonly, pulmonary artery narrowings occur within the first and/or second arcade branches. They present in one of three ways: (1) in conjunction with other forms of congenital heart disease (most commonly Tetralogy of Fallot), (2) in patients with well-defined syndromes such as Noonan, Williams Syndrome, or Alagille Syndrome [14, 15], or (3) as a consequence of surgery for congenital heart disease. The most common forms of iatrogenic pulmonary artery stenosis are from placement of aortopulmonary shunts and after repair of Tetralogy of Fallot, Transposition of the Great Vessels, and Truncus Arteriosus. Although pulmonary artery stenosis is a much more heterogenous disease with great anatomic variability, pulmonary artery stenosis is currently the most common indication for use of stents in the pediatric population. In particular in newborns and small children, treatment of this disease is limited only by the lack of an appropriate BAS for pediatric patients.

**Figure 3:**
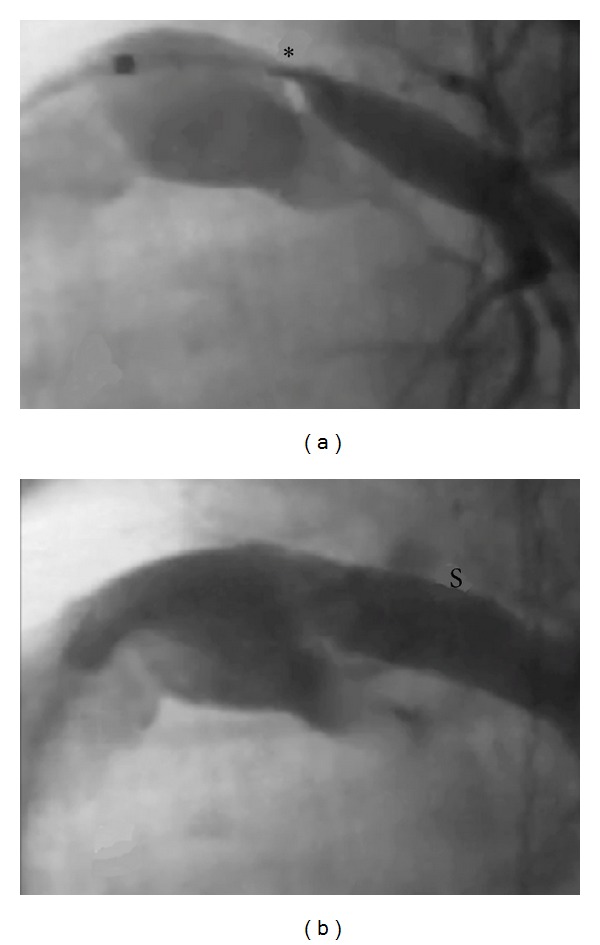
Infant with left PA stenosis before (a) and after (b) stent treatment. Although this provided effective relief of stenosis, this stent required serial redilation.

Similar to the situation with coarctation, use of BMS is currently considered to be the best treatment for pulmonary artery stenosis. Unfortunately, as with coarctation, use of BMS obligates children to serial stent redilation or even surgical removal in the future (Figure 3). Also, as with coarctation, results with angioplasty without stenting have been suboptimal. While proximal branch pulmonary arteries can be surgically repaired, many of the distal pulmonary arteries cannot be easily accessed and fixed surgically. Even repair of proximal pulmonary arteries requires an open chest operation to be performed on cardiopulmonary bypass with variable results and high rates of restenosis being observed in many centers.

BMS used to treat pulmonary artery stenosis is frequently complicated by “jailing off” side branches that are often crossed and blocked by metallic stents. A BAS would be an obviously superior stent for these situations as it could preserve side branches. A BAS designed for congenital applications would create a clearly superior device for the treatment of pulmonary artery stenosis in infants and smaller children.

There are two main categories of materials available for production of a stent that will degrade with time. One category is biocorrodable metal such as magnesium and iron. The other large category is bioabsorbable polymers [4]. We will begin by discussing polymers and the stents that have been produced from polymers to date. We will then discuss biocorrodable metals and the stents made from them. 

## 5. Composition: Polymers

### 5.1. Bioabsorbable Polymer Overview

A polymer is a large molecule made from many smaller units called monomers. The mechanical properties of a polymer are determined by many factors in addition to the monomer from which it is made. Properties such as stiffness, strength, and degradation time are affected by the number of monomers within the chain (molecular weight) and the arrangement of the monomers [16]. In general, the greater the molecular weight (longer chain of monomers), the greater strength and greater absorption time the polymer will have [16]. The chain structure also plays a large role in the strength of a polymer. A polymer consisting of multiple identical monomers in a linear sequence is the most simple and strongest type of polymer. Chains that are linear can pack themselves in a tight 3D arrangement, giving them great strength. This is referred to as high crystallinity. Nonlinear polymer chains have multiple branches, and the branches prevent the polymer from tightly packing. These highly branched polymers have a lower degree of crystallinity and less strength [16]. A polymer's properties are also affected by the chain's sequence isomerism, stereoisomerism, and addition of different end groups. Two different monomers can be combined to synthesize copolymers, which will have their own unique properties. Blending other materials with the polymers and adding catalysts to the polymer are additional methods available to manipulate polymer properties [16].

In addition to understanding a polymer's mechanical properties, it is important to understand how the polymer will degrade. All polymers degrade to some extent, but a polymer is only considered bioabsorbable if it is absorbed relatively quickly—during or immediately after its intended application [17]. The absorption process consists of two phases: degradation and erosion. Degradation is the process in which the polymer chain is cleaved into smaller oligomers and monomers. Bioabsorbable polymers consist of hydrolysable bonds, making hydrolysis the most important mode of degradation. Erosion is the process in which material is lost from the polymer as monomers and oligomers leave the polymer through pores and cracks created during degradation [17].

The rate of hydrolysis is determined in part by the class of bond linking the monomers together to form the polymer. Three common classes of bioabsorbable polymers are poly-anhydrides, polyorthoesters, and polyesters. They each have significantly different degradation half-lives. The *in vitro* half-life of polyanhydrides is less than 10 minutes, while the half-life of polyesters is 3.3 years [17]. The rate of each polymer's degradation can be altered. Altering the pH, adding enzymes, catalysts, hydrophilic or hydrophobic end groups, and adding a copolymer are all ways to adjust the degradation rate and half-life of the polymer so it better serves its intended application [17]. Models have been developed to simulate the mechanical behavior of bioabsorbable polymers as they degrade. Vieira et al. showed that the decrease in tensile strength of the copolymer PLA-PCA follows the same trend as the decrease in molecular weight, so a first order equation can be used to model the change in mechanical properties with degradation [18]. This model is helpful when choosing a polymer for bioabsorbable stent development. Once a material has been chosen and a prototype stent is made, extensive *in vitro* testing of the stent is performed. The stents are placed in saline baths mimicking an *in vivo* environment, and their thickness, weight, and radial strengths are measured over time. 

While the mechanical properties of polymers can be manipulated, it remains difficult to create a stent with the same radial force and limited recoil compared to metal stents. To achieve the necessary strength, polymer based stents have more bulk than metal stents, containing struts that are at least 150 *μ*m (microns) thick [9]. Polymer based stents have a larger crossing profile and require a larger sheath for delivery compared to metal stents [9]. As previously mentioned, a stent intended for use in babies needs to be low profile to avoid femoral arterial or venous damage from a large sheath. This may be one of the largest challenges in developing a polymer based bioabsorbable stent for use in the pediatric population.

### 5.2. Poly(L-Lactic Acid) PLLA

PLLA is the polymer most commonly used for the production of bioabsorbable stents. PLLA is an aliphatic polyester composed of the L-enantiomer of lactic acid (2-hydroxy propionic acid) [19]. It is a well-studied, high strength, thermoplastic polymer that can be processed with injection molding, blow molding, thermoforming, and extrusion applications [19]. The polymer has a glass transition temperature of 55 degrees Celsius. This is the temperature at which there is a reversible transition of a polymer from a relatively hard state into a rubber-like state. PLLA has a melting point of 175 degrees Celsius, and it must be heated to 185–190 degrees Celsius for processing. PLLA begins to lose molecular weight via chain scission reactions and thermal degradation when heated to 185 degree Celsius, so there is a narrow window of processing [19]. Addition of a small amount of the D-enantiomer improves the polymer's processability by depressing the melting point; however, this also decreases the polymer's crystallinity [19]. 

**Figure 4:**
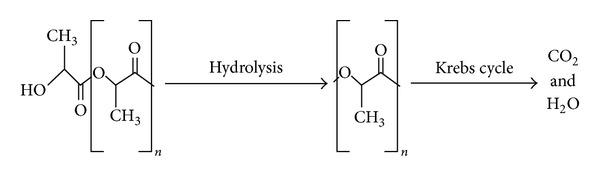
Hydrolysis of PLLA into lactic acid, which is further metabolized into carbon dioxide and water.

PLLA is degraded by simple hydrolysis of the ester bond, and it is then metabolized via the Krebs cycle into water and carbon dioxide [4, 5] (Figure 4). There are five stages of bioresorption, which can overlap. The first step is absorption of water from the surrounding tissue. The second phase is depolymerization by hydrolysis which results in a loss of molecular weight. The third phase is fragmentation of the polymer into segments of low-molecular weight polymer resulting in loss of mass. At this point the polymer no longer has radial strength. There is scission of chains linking the crystalline regions, and the shorter chains diffuse out of the device. The small polymers are engulfed by cells using phagocytosis and subsequently metabolized to L-lactate and converted to pyruvate. The pyruvate is broken down into carbon dioxide and water via the Krebs cycle [20]. The degradation time is dependent on molecular weight, crystallinity, temperature, impurities, and catalyst concentration [19]. Addition of the D-enantiomer to the polymer will alter the mechanical properties, processing temperatures and degree of crystallinity [19]. PLLA is soluble in organic solvents such as benzene, chloroform, and tetrahydrofuran [19]. PLLA of varying molecular weight and crystallinity is readily available for purchase from labs across the world. Companies such as Teleflex Medical OEM, Synterra, and Goodfellow sell PLLA online for anywhere from $300 to $500 per kilogram.

**Figure 5:**
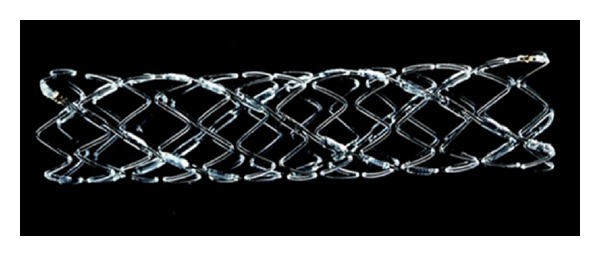
The Igaki-Tamai stent.

The first bioabsorbable stent placed in humans was the Igaki-Tamai stent (Figure 5). It is made of high molecular weight (321 KDa) PLLA, which is radiolucent and contains two radio-opaque gold markers [4, 21]. It has a zig-zag helical coils with straight bridges design which provides a stent-to-artery coverage ratio of 24%. Its strut thickness is 170 *μ*m [22]. It is a hybrid self-expanding stent that is mounted on a standard angioplasty balloon. The self-expansion requires heat. A delivery balloon filled with contrast heated to 70 degree Celsius triggers the initial expansion, and the final self-expansion occurs within 20 to 30 minutes of deployment when the stent is at body temperature. The FIM study demonstrated continued expansion of the stent for three months after deployment and no significant inflammatory response. The stent provides radial support for 6 months and appears completely absorbed by IVUS 2-3 years after deployment [23]. 

A second version of the stent was created which contained Tranilast, an antiproliferative tyrosine kinase inhibitor. This stent causes significantly less neointimal formation than the original stent [23]. The stent is not currently being used in coronary arteries, and this is likely secondary to the complicated delivery methods requiring heat for self-expansion. 

The Absorb Bioresorbable Vascular Scaffold (BVS) (Abbott, Santa Clara, CA) is the most advanced, well-studied bioresorbable vascular scaffold. Absorb is referred to as a scaffold to indicate its temporary nature in contrast to a permanent metallic stent. A small mesh tube, the BVS backbone, is made from poly(L-lactide) (PLLA), and it is covered with a layer of poly(D,L-lactide) (PDLLA) which releases everolimus, an antiproliferative medication. The scaffold is balloon expandable and has two platinum radio-opaque markers [4, 22]. The initial design, available in 3.0 mm diameter and 12 mm and 18 mm lengths, had a scaffold of circumferential out-of-phase zigzag hoops with straight and direct links. Its strut thickness was 158 *μ*m, the crossing profile was 1.4 mm, and the stent-to-artery coverage was 25% [22]. To retard physical aging of the polymer, the BVS was stored at −20 degrees Celsius and had a shelf life of 8 weeks [5]. It was implanted in 30 patients as part of the ABSORB Cohort A trial and was found to provide mechanical support to the coronary artery for a few weeks, while its degradation time was two years [24, 25]. During follow-up, patients were administered pharmacological agents aimed at assessing vessel motion, and preliminary evidence suggested that natural vessel motion was restored, which has been hypothesized to improve long-term clinical outcomes [26].

**Figure 6:**
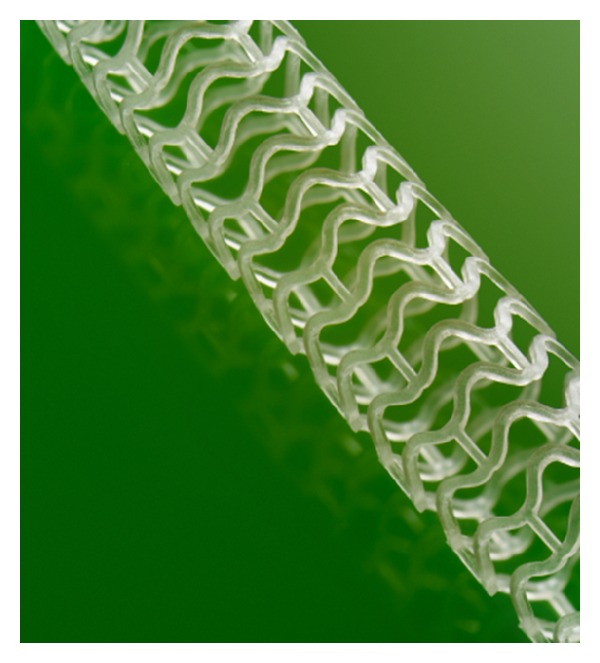
The Absorb Bioresorbable Vascular Scaffold.

Based on the knowledge generated during the Cohort A phase of the ABSORB trial, Abbott made modifications to the Absorb BVS focused on increasing the duration of vessel support without compromising other key design parameters. The product is now available in multiple sizes, including 2.5 mm, 3.0 mm, and 3.5 mm diameters and 12, 18, and 28 mm lengths [27]. (Figure 6). It fully resorbs within 3 years and provides mechanical support for up to 6–12 months [22, 28]. The product no longer requires subambient storage, an important feature for large-scale manufacturing and distribution. 

The BVS is produced in Abbott's Temecula, California manufacturing facility. PLLA resin is melted and extruded into thick-walled, small diameter tubes. These tubes are subsequently heated and expanded into thin-walled, larger diameter tubes, imparting the material properties essential to product performance. The tubes are laser cut into the scaffold pattern, platinum markers are added to provide radio-opacity, and the stent is crimped onto the balloon catheter delivery system. Product quality is assessed during each step of the manufacturing process [29].

Results from the second Cohort B phase of the ABSORB trial were recently presented at the 62nd Annual Scientific Session of the American College of Cardiology. The rate of major adverse cardiovascular events (MACE) in 101 patients was 10% at the 3-year follow-up, results that are similar to those of best-in-class drug eluting stents [30]. Abbott is currently engaged in several clinical trials around the world with enrollment targeting more than 12,000 patients [5]. Abbott's BVS is commercially available in Europe, the Middle East, and parts of Asia and Latin America. In January 2013, Abbott announced the initiation of ABSORB III, a randomized clinical trial of more than 2,000 patients that will support regulatory filings for approval of Absorb in the USA. 

It is hoped that the expertise obtained by Abbott in the manufacturing and testing of the BVS stent can be used to produce a larger diameter version acceptable for use in pediatric stenting. Abbott is now developing “peripheral” large diameter PLLA stents to target peripheral arterial disease. These devices could also be ideal for pediatric indications, but data is not yet available on these devices.

### 5.3. Tyrosine Polycarbonate Polymer

**Figure 7:**
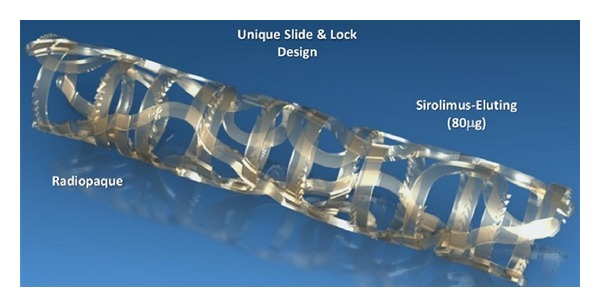
ReZolve Sirolimus-Eluting Bioresorbable Coronary Scaffold with slide and lock mechanism.

The REVA stent (Reva Medical Inc., San Diego, CA) is an absorbable stent made from a poly(desaminotyrosil-tyrosine ethyl ester) carbonate developed at Rutgers University [31]. It contains iodine to increase its radio-opacity [4, 5]. The tyrosine polycarbonate polymer is metabolized to water, carbon dioxide, ethanol, and the iodinated desaminotyrosyl-tyrosine. The iodinated amino acid is then absorbed and discreted [5]. The polymer has a slide and lock design which provides good radial force and flexibility as it can be expanded without material deformation (Figure 7). The strut thickness is 200 *μ*m, the crossing profile is 1.7 mm, and the stent-to-artery coverage is 65%. Delivery requires a 7F guide. Full degradation time for the stent is about three years [22]. When tested in the RESORB human trial, there was a high restenosis rate secondary to focal mechanical failures [4]. The polymer and slide-and-lock design was revised, and the updated ReZolve stent has improved strength and a coating of sirolimus [4, 5]. The ReZolve stent is currently being evaluated in the RESTORE study which was initiated in 2011 in Europe and Brazil [27]. Another redesign, the ReZolve 2 Scaffold, was developed and is undergoing initial testing (Figure 7). It utilizes an improved tyrosine polycarbonate polymer that provides a 30% increase in radial strength. It is also lower profile with a crossing profile of 1.52 mm and is 6F compatible [27]. Although the use of a poly(desaminotyrosil-tyrosine ethyl ester) carbonate for a pediatric BDS is provocative, the Reva stent design cannot be easily translated into a larger diameter stent design needed for pediatrics without a prohibitively large crossing profile. Although the “slide and lock” design provides excellent radial force, use of this material or stent design in pediatrics will require a major strut redesign.

### 5.4. Poly(Caprolactone) (PCL)

PCL is a polyester which has been used in bioabsorbable medical applications. It is a semi-crystalline polymer with good flexibility and a low melting point of approximately 60 degrees Celsius. It is nontoxic and causes minimal local inflammation during degradation. Compared to PLLA, PCL has less crystallinity and faster degradation times [31, 32]. A balloon expanding stent was designed from PCL at Chang Gung University which has a slide and self-locking mechanism, similar to the REVA stent previously discussed [33]. The stent consists of 2 elements, each an ellipse connected by two rings. The elements were produced by a lab-made microinjection molding machine. The stent elements were then interconnected, the assembly was rolled into a tube, and the connecting points were welded together with hot spot welding. The stents were then spray coated by a lab made device with poly(D,L)-lactide-coglycolide (PLGA) mixed with paclitaxel, an antiproliferative agent. *In vitro* testing showed that the stents had good compression strengths, similar to those of stainless steel stents, and there was minimal recoil after expansion. The compression strength and collapse pressures of the stent did not significantly decrease after 12 weeks in a solution designed to simulate the *in vivo* environment. The crystallinity of the PCL in the finished stent was lower than the crystallinity of the purchased PCL, suggesting that the cold mold processing technique decreased the polymer's crystallinity [33]. To our knowledge there has been no human trial with this stent. 

### 5.5. Poly(Anhydride Ester) Salicylic Acid

The IDEAL stent (Bioabsorbable Therapeutics Inc, Menlo Park, CA) is a balloon expandable stent made of salicylic acid (an anti-inflammatory agent) and a poly(anhydride ester). The stent back-bone polymer structure is repeating salicylate molecules. During absorption bonds between the salicylic acid and linker molecules are hydrolyzed and salicylic acid is released. The stent back-bone is covered in an 8.3 *μ*m thick coating of polymer containing sirolimus [1, 5]. The design is a tube with laser cut voids. The strut thickness is 200 *μ*m, the crossing profile is 2 mm, and the stent-to-artery coverage ratio is 65%. It provides radial support for 3 months, and the degradation time is 9 to 12 months. Salicylic acid was included to reduce local inflammation in addition to the antiproliferative properties of sirolimus. The WHISPER trial was the first human study. The stent provided good radial strength; however, significant neointimal growth was observed. A new stent with thinner struts, an improved design and a slower drug release is under development. The new design also has a lower profile making it 6F compatible [5, 34]. 

## 6. Composition: Biocorrodable Metal

While most of the work on bioabsorbable stents is being done with polymers, biocorrodable metals do have advantages over polymers. Stents made from biocorrodable metal tend to have greater strength and are lower profile compared to polymer based stents. In this way, they are very similar to traditional bare metal stents and present less of an engineering challenge compared to polymer based stents. We will discuss the most common biocorrodable metals and the stents produced from them to date. 

### 6.1. Magnesium Alloy

Magnesium is a biocompatible metal that corrodes in the body and combines with other elements to form magnesium oxide and organic salts. Magnesium's role in biological systems is well understood. 50% of the total body magnesium is found in bone, and it helps keep bones strong. It also supports a healthy immune system, helps maintain normal muscle and nerve function, and plays a role in maintaining a normal heart rhythm [9, 35, 36]. The amount of magnesium in currently developed stents ranges from 3 to 6 mg. The amount of released magnesium has almost no effect on the plasma magnesium concentration when absorbed over a period of months, and any increase in plasma magnesium would likely be beneficial rather than toxic [9]. Magnesium is antithrombogenic. It is also a systemic and coronary vasodilator which decreases systemic vascular resistance and increases the cardiac index [37]. Early experience with magnesium alloy stents suggest that the corrosion is well tolerated with very little associated inflammatory response [5, 9, 38]. An *in vitro* study by Sternberg et al. demonstrated that elevated magnesium levels in coronary artery endothelial and smooth muscle cells may actually have a beneficial effect in the setting of vessel injury. The data suggests magnesium decreases smooth muscle cell proliferation and increases endothelial cell proliferation [39]. 

The AMS-1 bioresorbable stent (Biotronik, Berlin, Germany) is a balloon expandable, radiolucent stent composed of 93% magnesium and 7% rare earth metals [5]. It is laser cut and polished from the WE-43 magnesium alloy tube into sinusoidal in-phase hoops liked by straight bridges. Strut thickness is 165 *μ*m, its crossing profile is 1.2 mm, it is 6F compatible, and its stent-to-artery coverage ratio is 10% [1, 22]. The fully expanded stent diameters are 3.0 and 3.5 mm, and the lengths are 10 mm and 15 mm [31]. It is radiolucent and does not contain radio-opaque markers. Clinical trials demonstrated it has good initial radial strength (0.8 bar), similar to traditional stainless steel stents; however, it only provides radial support for 1 to 2 weeks [4, 40]. In the PROGRESS AMS trial, the stent was almost undetectable by intravascular ultrasound four months after deployment. There were high rates of restenosis secondary to the very rapid degradation of the stent [41]. The stent was redesigned multiple times to increase degradation time and incorporate drug elution. The second design (Magic) used a different magnesium alloy (>90% magnesium, zirconium, yttrium, and rare earth metals), and different design with a square cross sectional shape of the strut and a reduced strut thickness of 120 *μ*m [22]. It is a premounted stent that is compatible with 6F introducer systems [9].

Research has shown that coating magnesium with biodegradable polymer is an effective method to slow magnesium's corrosion and loss of mechanical strength [41]. A study was performed comparing the degradation under cell culture condition of magnesium to magnesium covered in different types of biodegradable polymers [42]. The high molecular weight PLLA coated magnesium had a significantly lower corrosion rate compared to uncoated magnesium, and it showed the most uniform corrosion compared to low molecular weight PLLA coating and PCL coating. They concluded high molecular weight PLLA coating is a suitable option for slowing the corrosion of magnesium [42]. Another study showed PLLA and PCL can be applied to magnesium by spin coating. This study showed low molecular weight PLLA film had better adhesion strength to magnesium than the high molecular weight PLLA or PCL but concluded that both PLLA and PCLA are promising materials for protective coating [43].

**Figure 8:**
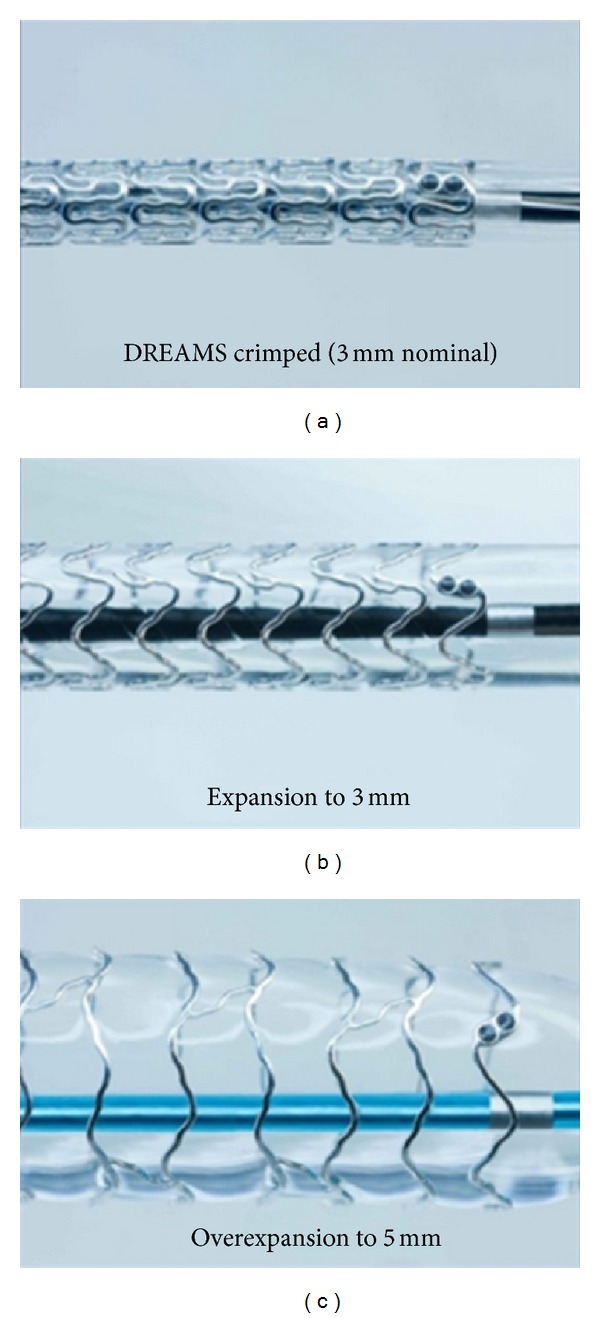
Biotronik DREAMS stent shown while crimped, expanded to 3 mm, and overexpanded to 5 mm.

The redesign of the AMS stent from Biotronik, the AMS-3 or DREAMS stent (Drug Eluting Absorbable Magnesium Scaffold), is covered with a layer of polymer to slow the degradation time and allow for Paclitaxel release (Figure 8). It is 6F compatible. The available diameters are 3 and 3.5 mm; however, it can be overexpanded up to 5 mm and still provide mechanical support [27]. During the first 3 months after implantation the polymer layer remains stable while the magnesium gradually begins to degrade. At 6 months the magnesium degradation is complete and the polymer absorption is ongoing. At 9 months the polymer is completely absorbed and structural disintegration begins. The Biosolve study is the first in-man study, and it showed improved mechanical properties with less early restenosis compared to the AMS-1 stent at 6-month follow-up [40, 44–46]. Biotronik is currently developing a second generation DREAMS stent that has an improved 6-crown 2-link design and is coated in PLLA carrying sirolimus. The new design provides greater radial stiffness and mechanical strength for a longer period of time. It also allows for increased postdilatation capabilities [27]. The radial stiffness of the 2nd generation DREAMS stent is comparable to metal stents, at 1.38 N/mm [27]. It contains radio-opaque markers and elutes sirolimus [27]. A clinical trial, BIOSOLVE-II, will commence in 2013 pending preclinical results [27].

More importantly, magnesium based biocorrodable stents are already being used in select cases in Europe for the treatment of pulmonary artery stenosis and aortic stenosis in infants. There are already several published case reports describing the use of magnesium stents in the pediatric population. Zartner et al. reported the first placement of a magnesium stent in the left pulmonary (LPA) artery of a 1.7 kg preterm baby born at 26-week gestation. The LPA had been inadvertently ligated, and a 3 mm × 10 mm AMS-I stent was successfully placed in the LPA in a hybrid fashion with a surgical cut-down of the pulmonary bifurcation. At 4-month follow-up the stent had completely degraded and the left lung continued to be well perfused. Despite the baby's small size, the stent was well tolerated without signs of local or systemic toxicity [47]. About 5 months after stent implantation the patient died from severe pneumonia. On autopsy the inner surface of the vessel was endothelialized with a smooth surface. The inner diameter of the lumen where the stent had been placed measured 3.7 mm. There were no visible or palpable pieces of the magnesium stent. Neointimal proliferation measured 100 *μ*m at its thickest, but there was no evidence of inflammatory reactions [2].

Schranz et al. reported the first use of a magnesium stent for treatment of coarctation of the aorta. A 15-day-old newborn developed severe long segment recoarctation after surgical coarctation repair. The baby was too unstable to tolerate repeat surgery. A 3.5 mm × 15 mm magnesium stent (AMS-1, Biotronik, Germany) was deployed with a 16 atm inflation which resulted in a final diameter of 4 mm. The coarctation reoccurred as the stent degraded, so a second magnesium stent was placed. Serum magnesium levels remained within normal limits even after placement of the second stent. At 3 months of age the baby had surgical closure of a ventricular septal defect. The aorta appeared widely patent at that time, but the surgeon decided to patch augment the previously stented aortic segment. For this reason no long-term follow-up is available [48]. 

### 6.2. Iron

Iron was the first metal used to make a biocorrodable stent. Iron stents have been placed in animals; however, none have reached clinical trials. The NOR-I stent (Devon Medical, Hamburg, Germany) is a balloon expandable stent that was placed in the descending aorta of New Zealand white rabbits [49]. Commercially available tubes consisting of 99.8% iron (Goodfellow, Cambridge, UK) were laser cut with a stent design similar to a stainless steel coronary stent (PUVA-AS16). To prevent corrosion, the tubes were laser cut in a nitrogen atmosphere. They were then electropolished to a strut thickness of 100–120 *μ*m. The expanded diameter of the stents ranged from 3 to 6 mm. No significant inflammation or neointimal formation was seen, there was minimal stent recoil, and there was no in-stent stenosis or thrombus formation at 18-month follow-up [49]. At 18 months the stent struts were still intact; however, there was some sign of degradation on histological evaluation [49]. 

A second similar iron stent was developed from 99.5% iron tubes purchased from Goodfellow Inc. (Huntingdon, UK). Additional contents of the tube included Aluminium, Calcium, Cobalt, Chromium, Copper, Mangenese, Nickel, Selenium, Carbon Phosphate, and Sulphur. The tubes were laser cut to a similar slotted tube design used in the Saxx stent (CR Bard, Tempe, AZ, USA) and were electropolished to a strut thickness of 120 *μ*m. The author's intention was to test an iron stent that could be implanted in a pediatric patient with congenital heart disease, so a larger stent diameter was chosen. The expanded diameters of the stents ranged from 6 mm to 12 mm. They were placed in the descending aortas of swine which were followed for up to one year. Results were similar to the NOR-I stent: the stents performed well without significant recoil or in-stent stenosis. There was no more local inflammation or neointimal formation compared to traditional stainless steel stents, and histopathological examination of all major organs showed no signs of iron related toxicity or overload. At one-year follow-up, large portions of the stent remained intact [50]. 

There are several advantages to using iron as a material for biocorrodable stents. Its mechanical properties are similar to those of traditional stainless steel stents. Iron is radio-opaque, so addition of markers to make the stent visible by fluoroscopy is not necessary. Compared to magnesium-based alloys, iron has a higher ductility so the laser cutting is less complicated. Iron is less brittle than magnesium, so iron stents can be made with thinner struts. The interaction of iron with the body, including its transportation and storage, is well understood. These stents contain only 40 mg of pure iron, about the recommended monthly intake of iron, so it should have good biocompatibility [51]. In the published animal studies, iron stents did not cause local or systemic toxicity from corrosion products, and they did not cause significant neointimal proliferation. They also performed as well as traditional stainless steel stents without significant restenosis. The iron stents degraded very slowly; however, design modification would be needed to expedite the degradation process for use in the pediatric population. 

Possible modifications include using iron based alloys, thinner struts, or a stent with designed areas of weakness resulting in stent fragmentation after endothelialization [49, 50]. A stent made from an iron-manganese alloy is being developed by powder metallurgy. *In vitro* studies show that this alloy has good mechanical properties and degrades faster than pure iron [52]. Electroformed iron is a material that may be very suitable for a bioabsorbable stent. Electroforming is a process that uses electrodeposition to produce metallic parts. The structure is formed atom layer by atom layer, so it is ideal for creating thin walled structures with dimensional precision [53]. A study was done to evaluate the feasibility of using electroformed iron to create a bioabsorbable stent. Flat sheets of electroformed iron were created, and the mechanical properties, microstructure, and corrosion behavior of these sheets were compared to pure iron and stainless steel. The electroformed foils measured 100 *μ*m thick and yielded strength comparable to that of stainless steel. It exhibited a higher corrosion rate compared to CTT-Fe pure iron, and a lower corrosion rate compared to magnesium. These properties make it a good candidate for application as a biocorrodable stent material [54].

A numerical model has been developed to predict the effect of corrosion on the mechanical properties of metallic bioabsorbable stents. Metallic foils were studied, but the model is applied in such a way to allow analysis of complex three-dimensional structures. The model is able to predict the performance of a bioabsorbable metallic stent as it corrodes over time [55].

## 7. Conclusion

There is a need for a bioabsorbable stent for use in children with congenital heart disease. We currently have absorbable stents for use in adult coronary artery disease which are not yet FDA approved. The largest expanded diameter of these stents is generally 4 mm. Treatment of coarctation of the aorta or pulmonary artery stenosis in a newborn would require a stent which can be expanded to 6–8 mm. The stent needs to be low profile to minimize vessel injury. While the bioabsorbable coronary stents are relatively low profile, a larger version of the same stent design that could be expanded to 8 mm would be higher profile. The stents need to be redesigned to maintain adequate radial strength and have a larger expanded diameter without a larger crossing profile. It needs to have the mechanical strength to prevent vessel recoil, while also absorbing in a short enough time to allow vessel growth. Development of absorbable coronary stents has led to a great understanding of the available materials and production techniques, and children with congenital heart disease will hopefully soon benefit from the current generation of bioabsorbable and biocorrodable materials and devices.
